# Cardiotoxicity After Synthetic Cathinone Use; Two Cases, A Case Series and Scoping Review

**DOI:** 10.1007/s12012-024-09832-x

**Published:** 2024-02-27

**Authors:** K. L. Groenewegen, F. M. J. Gresnigt, J. J. Nugteren-van Lonkhuyzen, C. den Haan, E. J. F. Franssen, R. K. Riezebos, D. Ohana, D. W. de Lange

**Affiliations:** 1 Resident Cardiology, Heartcenter, OLVG Amsterdam, Oosterpark 9, 1091 AC Amsterdam, The Netherlands; 2 Emergency Physician, Emergency Department, OLVG Amsterdam, Oosterpark 9, 1091 AC Amsterdam, The Netherlands; 3 Consultant Clinical Toxicology, Dutch Poisons Information Centre, UMC Utrecht, 3508 GA Utrecht, The Netherlands; 4 Dutch Poisons Information Centre, UMC Utrecht, 3508 GA Utrecht, The Netherlands; 5 Information Specialist, OLVG Amsterdam, Oosterpark 9, 1091 AC Amsterdam, The Netherlands; 6 Hospital Pharmacist-Clinical Pharmacologist and Toxicologist, OLVG Amsterdam, Oosterpark 9, 1091 AC Amsterdam, The Netherlands; 7 Heartcenter, OLVG Amsterdam, Oosterpark 9, 1091 AC Amsterdam, The Netherlands; 8 Currently, Cardiologist, Heartcenter Isala Zwolle, Dokter Van Heesweg 2, 8025 AB Zwolle, The Netherlands; 9 Center for Health Protection, National Institute for Public Health and Environment (RIVM), Antonie Van Leeuwenhoeklaan 9, 3721 MA Bilthoven, The Netherlands; 10 Toxicologist-Intensivist, Intensive Care and Dutch Poisons Information Centre, UMC Utrecht, 3508 GA Utrecht, The Netherlands

**Keywords:** Cardiovascular complications, Acute coronary syndrome, Arrhythmia, Out of hospital cardiac arrest, Synthetic cathinones, Designer drugs, New psychoactive substances

## Abstract

The cardiotoxic effects of synthetic cathinones remain largely unknown. In this study, we present two cases, a case series and a scoping review, to explore synthetic cathinone associated cardiotoxicity. Case 1 involved a 28-year-old male with non-ST-elevation myocardial infarction after ingesting a substance containing 4-methylmethcathinone (4-MMC), 3-methylmethcathinon (3-MMC), and methcathinone. Case 2 involved a 49-year-old male with ventricular fibrillation after 4-methylmethcathinone ingestion, who was diagnosed with severe three-vessel disease. A retrospective analysis was performed on self-reported synthetic cathinone poisonings reported to the Dutch Poisons Information Centre from 2012 to 2022. A total of 222 mono-intoxications with cardiotoxicity were included, mostly involving 3-methylmethcathinon (63%). Often tachycardia, hypertension, palpitations, and chest pain were reported. A comprehensive literature search was performed on PubMed to identify the studies reporting cardiac arrest, myocardial infarction, cardiac inflammation, cardiomyopathy, and life-threatening arrhythmias following synthetic cathinone use. A total of 30 articles reporting 40 cases were included. The reported complications included cardiac arrest (*n* = 28), ventricular tachycardia (*n* = 4), supraventricular tachycardia (*n* = 1), ST-elevation myocardial infarction (*n* = 2), non-ST-elevation myocardial infarction (*n* = 2), cardiomyopathy (*n* = 1), and myocarditis (*n* = 2). A total of ten different associated synthetic cathinones were identified. Cardiac arrest, myocardial infarction, and ventricular arrhythmias have been reported following the use of synthetic cathinones, underscoring the importance of obtaining a detailed recreational drug use history from patients presenting with syncope, chest pain, or palpitations.

**Supplementary Information:**

The online version contains supplementary material available at 10.1007/s12012-024-09832-x.

## Introduction

Synthetic cathinones are analogues of the *Chatha Edulis* plant (*Khat*)*,* which is used for its psychostimulant effects [1]. Synthetic cathinones augment monoamine transmission [2].This effectuates elevated intrasynaptic levels of dopamine, serotonin, and noradrenaline, either by inhibiting reuptake or by enhancing release of the monoamines [3, 4]. They structurally resemble methamphetamine [4]. Synthetic cathinones initially included 3,4-methylenedioxypyrovalerone (MDPV), 4-methylmethcathinone (mephedrone; 4-MMC), and 3,4-methylenedioxymethcathinone (methylone; MDMC) [5]. In recent years, the chemical structure has been altered in order to avoid legislation, resulting in at least 156 different types of synthetic cathinones [6]. In 2016, the five most commonly seized cathinones in Europe were alpha-pyrrolidinopentiophenone (α-PVP), 4-chloromethcathinone (4-CMC), 3-chloromethcathinone (3-CMC), 4-MMC, and 3-methylmethcathinone (3-MMC) [7].

Due to the changing pharmacodynamic profiles, many clinical effects are unknown, although cardiovascular, neurological, and psychopathological symptoms have been reported, including tachycardia, hyperthermia, insomnia, agitation, hallucinations, delusions, and confusion [3]. Since the use of *Khat* has been suggested as a risk factor for acute coronary syndrome, it can be expected that synthetic cathinones also cause cardiovascular complications [8]. With an increase in popularity of these new designer drugs and a variability of pharmacodynamic profiles, physicians face a challenge in recognizing and treating their side effects [6, 9].

The aim of this case series and scoping review was to investigate cardiotoxicity in association with the use of synthetic cathinones. Two exemplary cases, a case series from Poisons Information Centre data and an overview of previously reported cases on cardiotoxicity after synthetic cathinone use, are reported.

## Methods

### Cases

Blood was taken peripherally (brachial vein). Urine toxicology screening was performed using the Triage® TOX Drug Screen. Urinary toxicology screening was done with a panel of immunoassays (Triage, Alere). Comprehensive toxicological screening in serum was performed using the Toxtyper® a LC–MSn method with a Tox-library of prescription, over-the-counter and recreational drugs. The Toxtyper® is unable to differentiate between 3-MMC and 4-MMC. To distinguish the presence of 3-MMC and/or 4-MMC, samples were send to the Dutch National Institute for Public Health and environment (RIVM). First, a general screening was performed, using a Waters Acquity™ ultraperformance liquid chromatography (UPLC) system, to confirm the presence of 3-MMC or 4-MMC and possibly other active substances. Chromatographic and mass data were acquired and analyzed using Waters MassLynx v4.1 software. After the screening, the identity of 3-MMC and the absence of 4-MMC were confirmed and quantification was performed by an analysis on a Shimadzu Nexera X2 ultraperformance liquid chromatography system (UHPLC). Informed consent by both patients was granted.

### Case Series

The Dutch Poisons Information Centre provides a 24/7 telephone service for the management of acute poisonings, for health care professionals only, serving the entire Dutch population of 17.5 million inhabitants. During every telephone consultation, an electronic case record form is completed and stored in the center’s database. Anonymous data are routinely collected on patient (e.g., age and gender) and exposure characteristics (e.g., substance[s], reason for exposure), as well as on toxicity (symptoms present before or during the inquiry). The exposure data in the database generally lack analytical confirmation and are based on patient self-reported recreational drug use. For this study, a retrospective analysis was performed of electronic case record forms containing synthetic cathinone poisonings stored in the Dutch Poisons Information Centre data from 2012 to 2022 (11 years). To describe the cardiotoxicity of synthetic cathinones, poisonings with concomitant exposure to cocaine, amphetamine-type stimulants, heroin, and/or gamma-Hydroxybutyric acid were excluded. Data collected included the specific synthetic cathinone substance used and the cardiovascular symptoms reported during consultation.

### Review

For this scoping review, a literature search was performed (CH) through the electronic PubMed database from inception until 28-12-2022. Mesh- and TIAB key terms were used for the equivalents of brand and ‘street’ names of currently known synthetic cathinones, designer drugs, and new psychoactive substances (Supplementary information 1). These were combined with Mesh and TIAB key terms for equivalents of cardiac, heart disease, myocard, coronary, arrhythmia, ST-elevation myocardial infarction, and non-ST-elevation myocardial infarction. Title and abstract screening were performed (KLG), and potentially relevant articles were obtained for full-text review using a screening and selection tool (Supplementary information 2). All original observational studies (case reports, case series, case–control, and cohort studies) and interventional studies (randomized controlled trials and experimental studies) with self-reported and/or toxicologically confirmed synthetic cathinone use, presenting primarily with supraventricular and ventricular arrhythmia, myocarditis, cardiomyopathy, acute coronary syndrome, or cardiac arrest were included. Review articles, editorials, letters, animal studies, studies in languages other than Dutch or English, and cardiac arrest secondary to other symptoms (e.g., agitation, seizures, hyperthermia, renal failure) were excluded (Supplementary information 3). Also, co-intoxications with cocaine, amphetamine-type stimulants, heroin, and gamma-Hydroxybutyric acid were excluded for its individual cardiotoxic effects [10–12].

## Cases

### Case 1

**Fig. 1:**
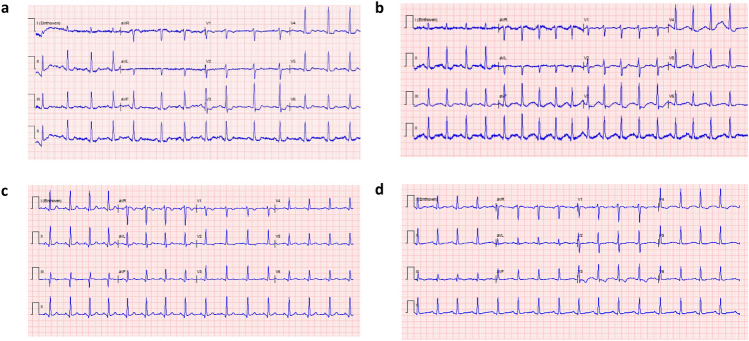
Electrocardiogram (ECG) of case 1 at different times during admission **a** 9:46 pm ECG at admission at the emergency room. **b** 10:06 pm ECG after administration of nitrates. **c** 1:19am ECG at admission on the cardiac care unit. **d** 8:04am ECG before discharge home

A 28-year-old male who had reported chest pain and confusion was found disoriented and motorically restless. Past medical history revealed Hemophilia A for which he used emicizumab. At the emergency department, the airway was uncompromised, and the oxygen saturation was 99% on room air, with a respiratory rate of 22 per minute. His blood pressure was 176/91 mmHg, with a heartrate of 91 beats per minute. Pupils were 4 mm, with isochoric reaction to light. No lateralization was observed, and his Glasgow Coma Scale score was 14. He was disoriented, highly associative, and repeatedly shouting words. Tympanic temperature was 37.3 °C. Self-reported history revealed ingestion of 2.5 ml *Alegria Forest Fruit* (SI 4)*,* two hours prior to the onset of symptoms, supposedly containing 3-CMC, 2-fluoroamphetamine (2-FA), 6-(2-Aminopropyl)benzofuran (6-APB) and 4-Hydroxy-N-methyl-N-ethyltryptamine (4-HO-MET), and also the ingestion of 4 to 6 units beer. He claimed to be a first-time recreational drug user and denied co-ingestion of other recreational drugs. Electrocardiogram (ECG) at admission showed a sinus rhythm of 90 beats per minute with ST-depression in V3 to V5 (Fig. 1a) that improved after administration of nitrates, although ST-depression in V3 persisted (Fig. 1b). At presentation, serum high-sensitive troponin-T was 16 ng/l and increased to 21 ng/l after one hour (SI 5). Venous blood gas analysis showed a respiratory alkalosis and a lactate of 4.6 mmol/L (SI 6). The patient was diagnosed with non-ST-elevated myocardial infarction (NSTEMI) and was admitted to the cardiac care unit for telemetric observation (Fig. 1c) and did not develop complications. The next day, he was asymptomatic. Quick-look ultrasound revealed a normal left and right ventricular function. The repolarization disturbances on the ECG at discharge (Fig. 1d), completely normalized, and follow-up Holter exam was normal. Extensive toxicological blood testing with the toxtyper® revealed 4-MMC and/or 3-MMC, and methcathinone. Quantitative analysis with UPLC revealed the presence of only 3-MMC with a serum concentration of 96 ng/l. Results were negative for the alleged content of *Alegria Forest Fruit;* 2-FA, 6-APB, and 4-HO-MET. Unfortunately, the original drug sample was not available for testing.

### Case 2

**Fig. 2:**
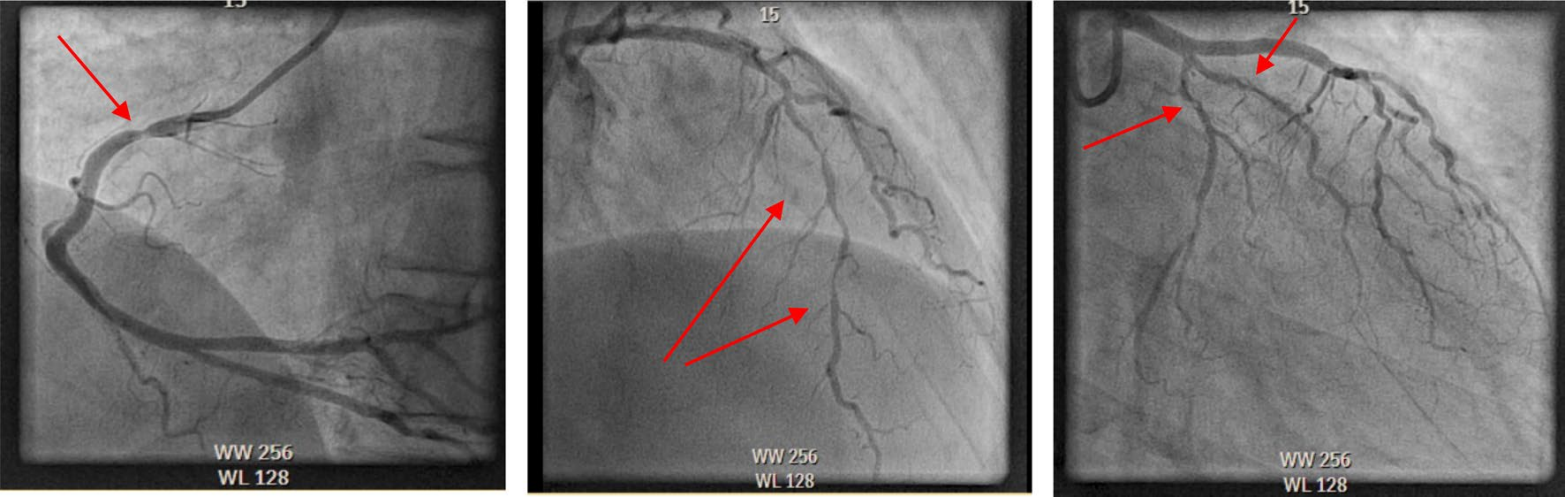
Electrocardiogram (ECG) of case 2 at different times during admission**.** **a** 8:00 pm ambulance ECG after return of spontaneous circulation. **b** 8:23 pm ECG at the emergency department. **c** 10:13 pm ECG at admission on the cardiac care unit

A 49-year-old male was found unconscious. Basic life support was started without delay. Quick-look rhythm showed ventricular fibrillation, for which he was defibrillated twice consecutively after which spontaneous circulation returned, without additional medication. The first ECG showed an atrial rhythm with a right bundle branch block and ST-depression infero-anteriorly (Fig. 2a). Two hours prior to collapse, the patient had taken 3-MMC and five units of alcohol and he had not mentioned any symptoms. Past medical history revealed an ST-elevation myocardial infarction (STEMI) eight years earlier of the anterolateral coronary artery for which he received a stent, with residual non-significant stenosis in the right coronary artery. The left ventricular function afterward was good. Other cardiovascular risk factors included non-active smoking (10 packyears) and familial hypercholesterolemia.

At the emergency department, the airway was uncompromised, and his oxygen saturation was 100% on 15L of oxygen via non-rebreathing mask, with a respiratory rate of 26 per minute, a blood pressure of 167/127 mmHg, a heart rate of 140 beats per minute, and a Glasgow coma scale score of 14, due to confusion. The first ECG in the emergency department showed a supraventricular tachycardia with minor ST-depression in the precordial leads (Fig. 2b). At the cardiac care unit, the ECG showed a sinus tachycardia with normal ST-segments (Fig. 2c). Serum high-sensitive troponin-T was elevated with a maximum of 837 ng/l (SI 7). Arterial blood gas analysis showed a respiratory acidosis with a pH of 7.26 and pCO2 of 52 mmHg (SI 8). Extensive toxicological screening in serum revealed the presence of 3-MMC and/or 4-MMC. Toxicology screening in urine did not reveal the presence of additional recreational drugs.

**Fig. 3:**
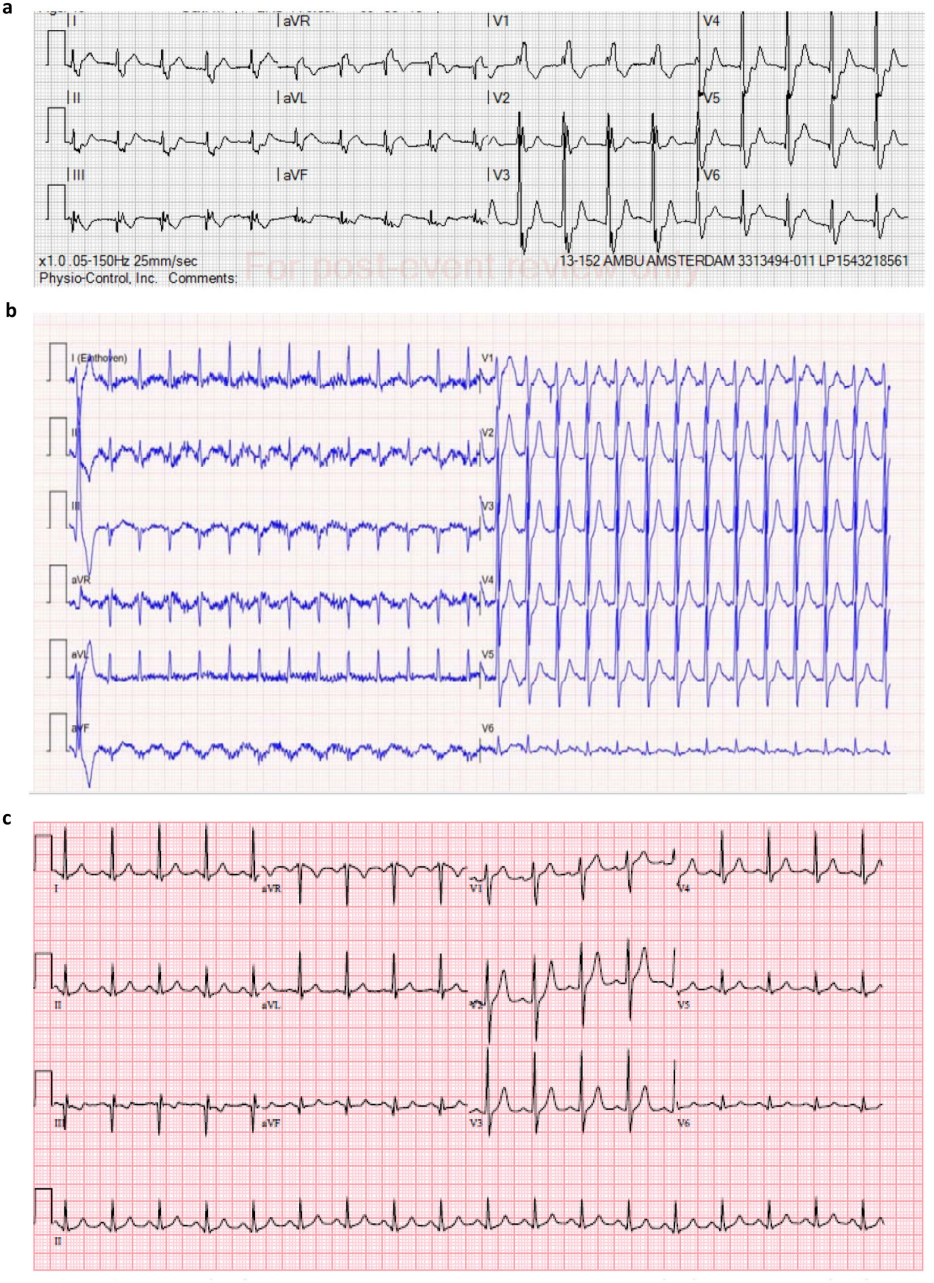
Coronary angiogram**.** Left image: 80% stenosis in the proximal right coronary artery. Middle image: diffuse calcifications with 90% stenosis in the left anterior descending artery. Right image: 80% stenosis in the proximal circumflex artery and 80% stenosis in the anterolateral artery

The patient was diagnosed with ventricular fibrillation due to recreational drug-induced non-ST-elevation myocardial infarction and was admitted at the cardiac care unit. Coronary angiography revealed severe three-vessel disease (Fig. 3). A quadruple coronary artery bypass grafting was performed, an implantable cardioverter-defibrillator (ICD) was implanted and further recovery was uncomplicated. He was discharged home after drug counseling.

## Results

### Case Series

In total, 222 synthetic cathinone poisonings (without relevant co-exposures) resulting in cardiotoxicity were reported by health care professionals to the Dutch Poisons Information Centre from 2012 to 2022 (Table 1). Most poisonings involved 3-MMC (63%), followed by 4-MMC (16%). Other synthetic cathinones were only reported incidentally (< 5%). Cardiovascular symptoms reported during consultation often involved tachycardia, hypertension, palpitations, and chest pain. In eight patients, ECG abnormalities were observed, including two patients with ventricular fibrillation requiring resuscitation after 3-MMC or 4-MMC use.

**Table 1:** Self-reported poisonings with synthetic cathinones and cardiotoxicity to the Dutch Poisons Information Centre

Specific substances	Cardiotoxicity *N* = 222
3-MMC	139 [63%]
4-MMC	36 [16%]
3-CMC	8 [4%]
Alpha-PVP	7 [3%]
4-MEC	4 [2%]
Hex-en	3 [1%]
MDPV	3 [1%]
3-CMC / 4-CMC	2 [1%]
Alpha-PHP	2 [1%]
Alpha-PiHP	2 [1%]
2-MMC	1 [< 1%]
ED-DB	1 [< 1%]
Ethylone	1 [< 1%]
MPHP	1 [< 1%]
Pentedrone	1 [< 1%]
Exposure to two synthetic cathinones*	11 [5%]
Symptoms	
Sinustachycardia (≥ 100 beats per minute)	139 [62%]
Sinus bradycardia (≤ 60 beats per minute)	2 [1%]
Hypertension (≥ 140 mmHg systolic or ≥ 90 mmHg diastolic)	69 [31%]
Hypotension (≤ 90 mmHg systolic or ≤ 60 mmHg diastolic)	7 [3%]
Palpitations	58 [26%]
Angina pectoris	56 [25%]
ECG abnormalities - prolonged QT-interval - ST-elevation - ST-depression - Negative T waves - Ventricular fibrillation	8 [4%] 2 2 1 1 2

* for details see *supplementary information SI 9*

### Review

A total of 30 articles were included, reporting 40 cases with cardiac arrest (*n* = 28), ventricular tachycardia (*n* = 4), supraventricular tachycardia (*n* = 1), ST-elevation myocardial infarction (*n* = 2), non-ST-elevation myocardial infarction (*n* = 2), cardiomyopathy (*n* = 1), and myocarditis (*n* = 2) after synthetic cathinone use (Table 2). Coronary atherosclerosis with thrombus or occlusion was found in eight cases, out of the 18 cases where coronary imaging or autopsy results were reported. Out of the six cases with a reported cause of cardiac arrest, one presented with ventricular fibrillation, three with pulseless electrical activity and two with asystole. Twenty-seven patients died, out of 35 cases with a reported outcome, mostly after sudden cardiac arrest (*n* = 16), of whom eight after a sudden collapse (three while exercising), five were found unconscious and three were found death. Nine other deceased patients presented to the emergency department with agitation or behavioral problems.

**Table 2:** Cases with cardiotoxic complication after synthetic cathinone use

Author	♀♂/Age	Synthetic cathinone	Route of exposure	Symptoms	Diagnosis and rhythm	Blood concentration	Co-intoxication	Relevant cardiac findings	Outcome
Beck et al.,2015 Sweden [49]	n.s	MDPV	n.s	n.s	Cardiac arrest	> 100 ng/mL	n.s	n.s	n.s
	n.s	MDPV	n.s	n.s	VT	n.s	–	–	n.s
Beck et al., 2016 Sweden [50]	♂ n.s	α-PVP	n.s	Agitation and delirium	Cardiac arrest	62.6 ng/mL	–	–	*Died*
Carbone et al., 2013 Canada [51]	♂ 19	Methylone	Unknown	Collapse while jogging	Cardiac arrest, PEA	0.07 mg/dL	–	*Autopsy: Bicuspid aortic valve, short right coronary artery, mild right ventricular dilatation*	*Died*
Cawrse et al., 2012 USA [52]	♂ 19	Methylone	n.s	Collapse while running	Cardiac arrest	0.67 mg/L (peripheral)	–	n.s	*Died*
Cherry and Rodriguez, 2017 USA [39]	♀ 42	α-PVP	Ingestion	Anterior STEMI	STEMI	n.s	n.s	CAG: Proximal thrombus total occlusion LAD, 80–90% stenosis proximal ramus intermedius, left ventricular apical thrombus	Survived
Chou et al., 2021 Taiwan [53]	♂ 37	N-ethylpentylone	Ingestion	Agitation and seizure	VT	n.s	25B-NBOMe	n.s	*Died*
deRoux and Dunn, 2016 USA [40]	♂ 30	Methylone	n.s	Collapse	Cardiac arrest	1.3 mg/L (femoral)	Ethanol 0.04 g%	n.s	*Died*
	♀ 25	Ethylone	n.s	Found unresponsive	Cardiac arrest	1.7 mg/L (femoral)	–	n.s	*Died*
	♂ 32	Methylone	n.s	Found dead	Cardiac arrest	0.64 mg/L (heart)	Ethanol 0.15 g%	n.s	*Died*
	♂ 37	Methylone	n.s	Found dead	Cardiac arrest	0.41 mg/L (cardiac)	n.s	Coronary artery thrombosis (not specified)	*Died*
	♂ 42	Methylone	n.s	Found dead	Cardiac arrest	0.6 mg/L (cardiac)	n.s	Coronary artery thrombosis (not specified)	*Died*
Desharnais et al., 2017 Canada [54]	♂ 42	*MDPV*	*Unknown*	Abnormal behavior	Cardiac arrest	*6 ng/mL (femoral and cardiac)*	*Mirtazapine, THC, 7-amino-clonazepam*	*Autopsy: Slight LVH*	*Died*
Eiden et al., 2013 France [13]	♂ 32	α-PVP	Snorting	Collapse	Cardiac arrest	1500 ng/mL (peripheral)	–	*Autopsy: atherosclerosis with 70% stenosis right coronary artery*	*Died*
Froberg et al., 2015 USA [36]	–	MDPV	n.s	Sympathomimetic symptoms	Cardiac arrest	82 ng/mL	Nicotine, cotinine, caffeine	–	*Died*
	–	MDPV	n.s	Sympathomimetic symptoms	SVT	< 10	Caffeine, hydrocodone, benzodiazepine, propofol	–	Survived
Fujita et al., 2016 Japan [55]	♂ 23	α-EAPP	Ingestion	Loss of consciousness with respiratory failure	Cardiac arrest	0.95ug/mL	Mepirapim	Autopsy: congestion of lungs and other organs	*Died*
Hobbs et al., 2022 USA [56]	♂ 30	4-fluoro-3methyl-α-PVP	Unknown	Unresponsive	VT	26 ng/mL (femoral)	–	Autopsy: cardiomegaly, dilated ventricles, bilateral pulmonary edema	*Died*
Ikeji et al., 2018 USA [57]	♂ 21	*N*-ethylpentylone	*unknown*	Combative, confused, sweating	Cardiac arrest	*n.s*	*Cannabis, ethanol*	–	*Died*
James et al., 2011 UK [58]	–	Mephedrone	n.s	Chest pain	(N)STEMI	n.s	n.s	n.s	n.s
	–	Mephedrone	n.s	Generalized convulsions	Cardiac arrest	n.s	n.s	n.s	n.s
	–	Mephedrone	n.s	n.s	Cardiac arrest	n.s	n.s	n.s	*Died*
Janiszewski et al., 2015 Poland [41]	♂ 26	Mephedrone	Snorting	Retrosternal chest pain	STEMI	n.s	Ethanol	CAG: total occlusion midsegment LAD	Survived
Kesha et al., 2013 USA [59]	♂ 37	MDPV	Ingestion	Delusion, agitation	VT, Cardiac arrest	0.7 mg/L (heart) 1.0 mg/L (peripheral)	Benzodiazepines, promethazine, salicylates, diphenhydramine	–	*Died*
Kovács et al., 2019 Hungary [42]	♂ 23	N-ethyl-hexedrone	n.s	Collapse while exercising	Cardiac arrest	285 ng/ml (phemoral)	ADB-FUBINACA (synthetic cannabinoid) 0.08 ng/ml	Autopsy: hypertrophic and dilated heart, severe atherosclerosis of the valves, coronary arteries	*Died*
Lee et al., 2022 Taiwan [60]	♂ 30	MDPV	n.s	Chest pain, cold sweats, dyspnea	Myocarditis	n.s	n.s	TTE: global hypokinesis with ejection fraction 20% and 1.5 cm-sized suspicious mass or vegetation in the left ventricle Myocardial perfusion imaging: mild inducible ischemia apex and basal inferolateral wall	Survived
Lenz et al., 2013 USA [37]	♂ 22	Mephedrone	Snorting	Dizziness, collapse	NSTEMI	n.s	–	Troponin 0.516 ng/L, normal ECG	Survived
Liveri et al., 2016 Cyprus [61]	♂ 42	MDPV Pentedrone	Unknown	Unresponsive	Cardiac arrest	0.046 mg/L (femoral) 0.16 mg/L (femoral)	Etizolam (0.3 mg/L), Ephedrine (0.068 mg/L), Olanzapine (4.2 mg/L), Mirtazapine (0.57 mg/L), Diclazepam	Autopsy: hypertrophic heart. No coronary atherosclerosis. Ischemic areas anterior and posterior wall left ventricle and septum	*Died*
Maskell et al., 2011 UK [43]	♀ 49	Mephedrone	Snorting	1. Chest pain, vomitus and collapse	Cardiac arrest	0.98 mg/L (femoral)	Cannabis, ethanol	Autopsy: old atherosclerotic occlusion proximal anterior descending artery, and myocardial fibrosis in anterior left ventricular wall. Histopathology: diffuse fibrosis, no acute ischemia	*Died*
	♂ 19	Mephedrone	n.s	2. Choking	Cardiac arrest	2.24 mg/L (femoral)	3-TFMPP, ethanol	–	*Died*
	♀ 55	Mephedrone	n.s	3. Unresponsive	Cardiac arrest	0.13 mg/L	Diazepam, nordiazepam, olanzapine, chlorpromazine, methadone (0.3 mg/L), EDDP, procyclidine, putrefactants	Autopsy: focal single atherosclerosis	*Died*
Murray et al., 2012 USA [62]	♂ 40	MDPV	Injection and Snorting	Agitation, delusional	Cardiac arrest, PEA	82 ng/mL	Ethanol (11 mg/dL), acetaminophen, cotinine, lidocaine, trimethoprim (12 mcg/mL)	–	*Died*
Nakamura et al., 2022 Japan [63]	♂ 32	Euthylene	Unknown	Abnormal behavior	Cardiac arrest, PEA	2500 ng/g (peripheral)	Aripiprazole (26.7 ng/g)	–	*Died*
Nicholson et al., 2010 Ireland [64] [51]	♂ 19	Mephedrone	Ingestion	Chest pain	Myocarditis	n.s	–	Cardiac MRI: anterolateral myocardial edema consistent with acute inflammation	Survived
Nugteren-van Lonkhuyzen et al., 2022 The Netherlands* [9]	–	3-MMC	Unknown	Agitation	Cardiac arrest, VF	n.s	Caffeine	–	Survived
Potocka-Banas et al., 2016 Poland [14]	♂ 28	α-PVP	n.s	Sudden arrest	Cardiac arrest	174 ng/mL (peripheral)	–	Autopsy: cardiac hypertrophy with a 2.5 × 1 mm ischemic scar	*Died*
Sellors et al., 2014 Australia [65]	♂ 44	α-PVP	Injection	Agitation, abnormal behavior	Cardiac arrest, asystole	n.s	–	–	*Died*
Sivagnanam et al., 2013 USA [66]	♂ 27	MDPV	Inhaling and injection	Agitation with mild hypotension (90/60 mmHg)	Cardiomyopathy with global hypokinesia	n.s	–	TTE: dilated cardiomyopathy with an EF of 15–20% and global hypokinesia. CAG: normal	Survived
Sykutera et al., 2015 Poland [15]	♂ 28	α-PVP Pentedrone	Unknown	Unconscious	Cardiac arrest, asystole	901 ng/mL (femoral) 8794 ng/mL (femoral)	–	Autopsy: moderately advanced atherosclerotic lesions of arteries. Histology: changes in the heart and presence of heart failure cells	*Died*
Weng et al. 2022 Taiwan [67]	–	n.s	n.s	n.s	Cardiac arrest	n.s	n.s	–	Unknown

*MDPV* methylenedioxypyrovalerone, *VT* ventricular tachycardia, *α-PVP α*-*pyrrolidinopentiophenone*, *PEA* pulseless electrical activity, *STEMI* ST-elevation myocardial infarction, *CAG* coronary angiography, *LAD* left anterior descending artery, 25B-NBOMe = 2-(4-bromo-2,5-dimethoxyphenyl)-N-(2-methoxybenzyl)ethanamine, *THC tetrahydrocannabinol*, *LVH* left ventricular hypertrophy, *α-EAPP* α- ethylaminopentiophenone, *NSTEMI* non-ST-elevation myocardial infarction, *TTE* transthoracic echocardiogram, *3-TFMPP 3*-trifluoromethylphenylpiperazine, *EDDP* 2-ethylideen-1,5-dimethyl-3,3-fenylpyrrolidine, *3-MMC* 3-methylmethcathinone, *n.s.* not specified,− = negative

*case also included in case series. For more detailed case description, see supplementary information 10

The types of synthetic cathinones reported in these cases were 3,4-methylenedioxypyrovalerone (MDPV), 4-methylmethcathinone (mephedrone; 4-MMC), 3-methylmethcathinone (3-MMC), pyrrolidinopentiophenone (α-PVP), 3,4-methyleendioxymethcathinon (methylone), N-ethylpentylone, α-ethylaminopentiophenone (α-EAPP), N-ethyl-hexedrone, ethyl-pentedrone, 3,4-methylenedioxy-N-ethylcathinone (euthylone), and 3-methoxy-2-(methylamino)-1-(p-tolyl)propan-1-one (mexedrone). In 18 cases, the toxicological screening revealed co-intoxication, with mostly ethanol (*n* = 7), cannabis (*n* = 3), or synthetic cannabinoids (*n* = 2), but also nicotine, caffeine, benzodiazepines, methadone, antidepressants, and antipsychotics were reported.

## Discussion

Two new cases of cardiotoxicity after synthetic cathinone use were presented, one with acute coronary syndrome after 3-MMC use, and one with cardiac arrest after 3-MMC and/or 4-MMC use. Extensive toxicological screening did not reveal other stimulant or sympathomimetic drugs such as cocaine, MDMA, or 4-FA. Another 225 cases were reported to the Dutch Poisons Information Centre with self-reported mono-intoxication with synthetic cathinones. Most poisonings involved 3-MMC (62%) or 4-MMC (16%) and cardiovascular symptoms mostly reported were tachycardia, hypertension, palpitations, and chest pain. Two patients developed ventricular fibrillation. Besides these new cases, 40 additional cases with cardiovascular complications after synthetic cathinone use were identified.

Interestingly, the analytical test in case 1 revealed a substance different from what the patient claimed to have taken, suggesting that he may have received substances different from those he purportedly purchased. This discrepancy was observed in a few other reported cases [13–15]. The issue of drug contamination, misleading information on packages, or disinformation provided by street sellers is a well-known problem. A retrospective analysis of hair samples confirmed the presence of synthetic cathinones in users who claimed to have used substances other than synthetic cathinones [16–18]. In 3-MMC samples collected by the Dutch National Institute of Mental Health and Addiction, different substances were often detected, such as 3-CMC or 2-MMC [19]. Consequently, this can lead to unexpected and undesired interactions and side effects among users. Most patients reported to the Dutch Poisons Information Centre lacked toxicological confirmation, making it possible that other substances may be responsible for these cardiovascular symptoms and complications.

4-MMC and 3-MMC are both synthetic cathinones with a similar chemical structure. 3-MMC was synthesized around 2010 to replace 4-MMC, which was criminalized in several countries [20]. The synthesis of new psychoactive substances is an ongoing process in reaction to the illegalization of specific designer drugs. Additionally, 3-CMC and 2-MMC have been identified [3, 21]. Pharmacologically, both 4-MMC and 3-MMC inhibit the reuptake of noradrenaline, compared to 4-MMC, 3-MMC exhibits more potent dopamine inhibitory effects than serotonin, and both substances induce a sympathomimetic toxidrome. In 3-MMC, tachycardia and agitation were predominantly reported, while among 4-MMC users, diaphoresis, headache, palpitations, and nausea were most commonly reported [20, 22, 23].

Synthetic cathinones bear a resemblance to the chemical structure of amphetamine-type stimulants through the substitution of an alkyl or halogen structure [2]. Pharmacodynamically, amphetamine-type stimulants, as well as synthetic cathinones, bring about higher intrasynaptic levels of dopamine, noradrenaline and serotonin [24]. However, the precise interaction of synthetic cathinones with the cardiovascular system remains unknown. A possible mechanism by which synthetic cathinone affect the cardiovascular system was recently reviewed by Radaelli et al. [25]. In rats injected with 4-MMC, an increased stroke volume, cardiac output, and contractility were observed, suggesting a potential cause of cardiac ischemia and death [26]. Furthermore, an impairment in mitochondrial function was considered, leading to oxidative stress and subsequently cardiomyocyte apoptosis [25]. Additionally, altered potassium, sodium, and calcium channels could contribute to the occurrence of arrhythmias [25].

Long-term cardiovascular effects of synthetic cathinones are unknown, but may be comparable to those after chronic *Khat* use: hypertension, coronary vasospasm, myocardial infarction, stroke, and heart failure [27–29]. A few cohort studies on acute cardiovascular effects after synthetic cathinone use have been performed, such as a poison control study with three months follow-up among 34 Hagigat (benzoylethanamine) users, reporting three (8.8%) young patients (16, 25 and 26 years old) with non-ST-elevation myocardial infarction [30]. Another small (*n* = 8) prospective poison control study reporting on 3-MMC users, reported tachycardia, severe hypertension (systolic BP > 180 mmHg), and cardiac arrest as adverse effects [9]. Together with all the cases reported in this review, this strongly suggests that synthetic cathinones induce cardiovascular complications and this should be taken into account by physicians treating patients with synthetic cathinone associated cardiovascular symptoms.

Because cocaine and amphetamine-type stimulants are known for their increased risk for cardiovascular complications, combined use with these substances was excluded from this case series and literature review[10–12]. Also GHB and heroine were excluded, since both drugs are mostly known for their depressive effects on the central nervous system, resulting in bradypnea or apnea. Therefore, the cause of cardiorespiratory arrest is unclear in a co-intoxicated patient with both cathinones and GHB or heroine [31, 32]. Furthermore, altered sympathicomimetic cardiovascular response has been described after GHB use [33]. Nevertheless, combination of synthetic cathinones with other sympathomimetic drugs might increase the risk of developing cardiovascular complications as well, although this effect has not been described so far. Although there is some evidence that simultaneous use of 4-MMC and ethanol can further increase the blood pressure and heart rate [34]. Since co-intoxication is not uncommon among recreational drug-users, users should be warned for potential additional risks [10–12].

In the United States, chest pain is the second most common complaint in the emergency department, and awareness of a potential toxicological origin for chest pain is important [35]. In these patients, a full cardiologic workup according to local guidelines is indicated to detect myocardial ischemia. In the first case described, slightly elevated cardiac enzymes were found with ST-depression on the ECG, which was reported previously after MDPV and 4-MMC use and was classified as non-ST-elevation myocardial infarction [36, 37]. Nevertheless, interpreting cardiac enzymes can be challenging after recreational drug use, since troponin release is triggered not only by ischemia, but also by extensive physical activity and psycho-emotional stress, like cathinone induced noradrenergic effects combined with agitation [38]. To adequately interpret these troponin levels, a drug use history in every chest pain patient is essential.

Coronary atherosclerosis with thrombus or occlusion was found in eight cases, out of the 18 cases where coronary imaging or autopsy results were reported [13, 15, 39–43]. This might be due to a cathinone-induced increased coagulability. In a cohort study among 146 patients, the INR was on average 0.2 points lower in *Kath*-chewers compared with non-*Kath*-chewers [44]. Nevertheless, 10 out of the 18 cases did not show coronary atherosclerosis, suggesting other causes may play a role, like the sympathomimetic effects or coronary vasospasm that was previously described in guinea-pigs, possibly induced by the noradrenergic effect of cathinones [45]. This supports the need for full cardiologic workup for patients with cardiovascular symptoms after synthetic cathinone use.

There is no antidote in the treatment for synthetic cathinone toxicity. For the sympathomimetic effects, agitation and behavioral problems, sedations with benzodiazepines or droperidol is recommended [46]. For the increased coagulability, antiplatelet therapy might be beneficial, although no evidence exist to support this. For acute arrhythmias and cardiac arrest, local resuscitation guidelines should be followed. Further treatment recommendation are according to the European society of cardiology guidelines, recommending toxicologic testing for newly documented ventricular arrhythmias, idiopathic ventricular arrhythmias, and sudden cardiac deaths of unknown etiology [47]. Also for acute coronary syndrome and cardiac arrest survivors, a non-coronary cause like intoxication should be ruled out [48]. It is our recommendation to include recreational drug use in all guidelines regarding cardiovascular complications and that physicians always provide drug counseling and referral for drug abuse treatment, if appropriate.

This study has several limitations, first of all in the reviewed cases, many data are missing, for example co-intoxications. Second, the self-reported drug use history may be unreliable, or the exact substance used may be uncertain, due to different street names (such as ‘bathsalts’ or ‘coffee packs´) and unreliable drug dealers, which was also true for the patient in case 1 and several reported cases from the literature review. In the cases reported in the review, toxicological analyses were performed to confirm the involved substance, but the type of sample (e.g., femoral or cardiac blood, urine) and type of confirmation tests were variable. Third, the causality for synthetic cathinone use and cardiovascular complication is uncertain, especially for the cases who were found dead. And in some cases, the cause of death was uncertain and could have been cardiovascular by nature, but also have another cause (e.g., respiratory failure, convulsions, hyperthermia). Fourth, multiple forms of reporting bias are expected, especially in view of the high number of fatalities found. Fifth, no conclusions can be drawn regarding the true prevalence of synthetic cathinone-associated cardiovascular complications. Besides the previously mentioned reporting bias, physicians may not question their patients on recreational drug use or report on this. These limitations should be taken into account before drawing conclusions regarding cardiotoxicity due to synthetic cathinone use, for which larger studies are necessary.

## Conclusion

In this study, two new cases illustrating cardiovascular complications following the use of synthetic cathinones were presented, accompanied by a case series comprising 222 patients reported to the Dutch Poisons Information Centre, and a review of existing literature, which identified an additional 40 cases. The documented cardiovascular complications included tachycardia, severe hypertension, supraventricular tachycardia, ventricular fibrillation, acute coronary syndrome, and cardiac arrest. The importance of recognizing the potential cardiotoxicity associated with synthetic cathinones should be emphasized. Therefore, healthcare providers should prioritize gathering a detailed recreational drug use history and obtaining toxicological confirmation in order to enhance awareness and ensure appropriate management, including drug counseling.

### Supplementary Information

Below is the link to the electronic supplementary material.

Supplementary file1 (DOCX 658 KB)

## References

[1] Silva B, Soares J, Rocha-Pereira C, Mladěnka P, Remião F, Researchers, O. B. O. T. O (2022). Khat, a cultural chewing drug: A toxicokinetic and toxicodynamic summary. Toxins.

[2] Simmons SJ, Leyrer-Jackson JM, Oliver CF, Hicks C, Muschamp JW, Rawls SM, Olive MF (2018). DARK classics in chemical neuroscience: Cathinone-derived psychostimulants. ACS Chemical Neuroscience.

[3] Wojcieszak J, Kuczyńska K, Zawilska JB (2020). Four synthetic cathinones: 3-chloromethcathinone, 4-chloromethcathinone, 4-fluoro-α-pyrrolidinopentiophenone, and 4-methoxy-α-pyrrolidinopentiophenone produce changes in the spontaneous locomotor activity and motor performance in mice with varied profile. Neurotoxicity Research.

[4] Loftis JM, Janowsky A (2014). Neuroimmune basis of methamphetamine toxicity. International Review of Neurobiology.

[5] Riley AL, Nelson KH, To P, López-Arnau R, Xu P, Wang D, Wang Y, Shen H-W, Kuhn DM, Angoa-Perez M, Anneken JH, Muskiewicz D, Hall FS (2020). Abuse potential and toxicity of the synthetic cathinones (i.e., ‘Bath salts’). Neuroscience and Biobehavioral Reviews.

[6] European Drug Report 2021: Trends and Developments. (2021). *European Monitoring Centre for Drugs and Drug Addiction (2021)*, *Publicatio*

[7] European Drug Report 2018: Trends and Developments. (n.d.). *European Monitoring Centre for Drugs and Drug Addiction (2018)*, *Publicatio*. 23787082

[8] Gresnigt FMJ, Smits ES, Den Haan C, Riezebos RK, Franssen EJF, De Lange DW (2022). The association of amfetamines and cathinones with acute coronary syndrome—A systematic review. Clinical Toxicology.

[9] Nugteren-van Lonkhuyzen JJ, Essink S, Rietjens SJ, Ohana D, de Lange DW, van Riel AJHP, Hondebrink L (2022). 3-Methylmethcathinone (3-MMC) poisonings: acute clinical toxicity and time trend between 2013 and 2021 in the Netherlands. Annals of Emergency Medicine.

[10] Murphy CM, Dulaney AR, Beuhler MC, Kacinko S (2013). ‘Bath salts’ and ‘plant food’ products: The experience of one regional US poison center. Journal of Medical Toxicology : Official Journal of the American College of Medical Toxicology.

[11] Thirakul P, Hair SL, Bergen LK, Pearson MJ (2017). Clinical presentation, autopsy results and toxicology findings in an acute N-ethylpentylone fatality. Journal of Analytical Toxicology.

[12] Roberts L, Ford L, Patel N, Vale JA, Bradberry SM (2017). 11 analytically confirmed cases of mexedrone use among polydrug users. Clinical Toxicology (Philadelphia, Pa.).

[13] Eiden C, Mathieu O, Cathala P, Debruyne D, Baccino E, Petit P, Peyriere H (2013). Toxicity and death following recreational use of 2-pyrrolidino valerophenone. Clinical Toxicology.

[14] Potocka-Banaś B, Janus T, Majdanik S, Banaś T, Dembińska T, Borowiak K (2017). Fatal intoxication with α-PVP, a synthetic cathinone derivative. In Journal of Forensic Sciences.

[15] Sykutera M, Cychowska M, Bloch-boguslawska E (2015). A fatal case of pentedrone and a -pyrrolidinovalerophenone poisoning. Journal of Analytical Toxicology.

[16] Palamar JJ, Salomone A, Vincenti M, Cleland CM (2016). Detection of “bath salts” and other novel psychoactive substances in hair samples of ecstasy/MDMA/“Molly” users. Drug and Alcohol Dependence.

[17] Salomone A, Palamar JJ, Gerace E, Di Corcia D, Vincenti M (2017). Hair testing for drugs of abuse and new psychoactive substances in a high-risk population. Journal of Analytical Toxicology.

[18] Rust KY, Baumgartner MR, Dally AM, Kraemer T (2012). Prevalence of new psychoactive substances: A retrospective study in hair. Drug Testing and Analysis.

[19] Hutten N, S. R. L. (2023). *Drugs Informatie en Monitoring Systeem (DIMS); Jaarbericht 2022*. Trimbos Instituut, Utrecht. https://www.trimbos.nl/wp-content/uploads/2023/05/INF143-DIMS-Jaarbericht-2022.pdf

[20] Bäckberg M, Lindeman E, Beck O, Helander A (2015). Characteristics of analytically confirmed 3-MMC-related intoxications from the Swedish STRIDA project. Clinical Toxicology.

[21] Nycz JE, Pazdziorek T, Malecki G, Szala M (2016). Identification and derivatization of selected cathinones by spectroscopic studies. Forensic Science International.

[22] Luethi D, Kolaczynska KE, Docci L, Krähenbühl S, Hoener MC, Liechti ME (2018). Pharmacological profile of mephedrone analogs and related new psychoactive substances. Neuropharmacology.

[23] Dargan PI, Sedefov R, Gallegos A, Wood DM (2011). The pharmacology and toxicology of the synthetic cathinone mephedrone (4-methylmethcathinone). Drug Testing and Analysis.

[24] Carvalho M, Carmo H, Costa VM, Capela JP, Pontes H, Remião F, Carvalho F, de Bastos ML (2012). Toxicity of amphetamines: An update. Archives of Toxicology.

[25] Radaelli D, Manfredi A, Zanon M, Fattorini P, Scopetti M, Neri M, Frisoni P, D’Errico S (2021). Synthetic Cannabinoids and cathinones cardiotoxicity: Facts and perspectives. Current Neuropharmacology.

[26] Meng H, Cao J, Kang J, Ying X, Ji J, Reynolds W, Rampe D (2012). Mephedrone, a new designer drug of abuse, produces acute hemodynamic effects in the rat. Toxicology Letters.

[27] Geta TG, Woldeamanuel GG, Hailemariam BZ, Bedada DT (2019). Association of chronic khat chewing with blood pressure and predictors of hypertension among adults in gurage zone, Southern Ethiopia: A comparative study. Integrated Blood Pressure Control.

[28] Ali WM, Zubaid M, Al-Motarreb A, Singh R, Al-Shereiqi SZ, Shehab A, Rashed W, Al-Sagheer NQ, Saleh AH, Al Suwaidi J (2010). Association of khat chewing with increased risk of stroke and death in patients presenting with acute coronary syndrome. Mayo Clinic Proceedings.

[29] Al-Motarreb A, Al-Kebsi M, Al-Adhi B, Broadley KJ (2002). Khat chewing and acute myocardial infarction. In Heart (British Cardiac Society).

[30] Bentur Y, Bloom-Krasik A, Raikhlin-Eisenkraft B (2008). Illicit cathinone (‘Hagigat’) poisoning. Clinical Toxicology (Philadelphia, Pa.).

[31] Busardò FP, Jones AW (2015). GHB pharmacology and toxicology: Acute intoxication, concentrations in blood and urine in forensic cases and treatment of the withdrawal syndrome. Current Neuropharmacology.

[32] Kiyatkin EA (2019). Respiratory depression and brain hypoxia induced by opioid drugs: Morphine, oxycodone, heroin, and fentanyl. Neuropharmacology.

[33] Hicks AR, Varner KJ (2008). Cardiovascular responses elicited by intragastric administration of BDL and GHB. Journal of Receptor and Signal Transduction Research.

[34] Papaseit E, Pérez-Mañá C, de Perna SFEB, Olesti E, Mateus J, Kuypers KP, Theunissen EL, Fonseca F, Torrens M, Ramaekers JG, de la Torre R, Farré M (2019). Mephedrone and alcohol interactions in humans. Frontiers in Pharmacology.

[35] CDC. (2016). *National Hospital Ambulatory Medical Care Survey EMERGENCY DEPARTMENT FACT SHEET*. https://www.cdc.gov/nchs/data/nhamcs/factsheets/2016_NHAMCS_ED_Fact_Sheet-508.pdf

[36] Froberg BA, Levine M, Beuhler MC, Judge BS, Moore PW, Engebretsen KM, Mckeown NJ, Rosenbaum CD, Young AC, Rusyniak DE (2015). Acute methylenedioxypyrovalerone toxicity. Journal of Medical Toxicology : Official Journal of the American College of Medical Toxicology.

[37] Lenz J, Brown J, Flagg S, Oh R, Batts K, Ditzler T, Johnson J (2013). Cristalius: A case in designer drugs. Military Medicine.

[38] Chaulin A (2021). Cardiac troponins: Contemporary biological data and new methods of determination. Vascular Health and Risk Management.

[39] Cherry SV, Rodriguez YF (2017). Synthetic Stimulant reaching epidemic proportions: Flakka-induced ST-elevation myocardial infarction with intracardiac thrombi. In Journal of cardiothoracic and vascular anesthesia.

[40] de Roux SJ, Dunn WA (2017). ‘Bath Salts’ the New York City medical examiner experience: A 3-year retrospective review. Journal of Forensic Sciences.

[41] Janiszewski M, Strojek M, Syska-Sumińska J, Dłużniewski M, Kuch M (2015). ST elevation myocardial infarction in a 26-year-old man after the use of mephedrone. Kardiologia Polska.

[42] Kovács K, Kereszty É, Berkecz R, Tiszlavicz L, Sija É, Körmöczi T, Jenei N, Révész-Schmehl H, Institóris L (2019). Fatal intoxication of a regular drug user following N-ethyl-hexedrone and ADB-FUBINACA consumption. Journal of Forensic and Legal Medicine.

[43] Maskell PD, De Paoli G, Seneviratne C, Pounder DJ (2011). Mephedrone (4-methylmethcathinone)-related deaths. Journal of Analytical Toxicology.

[44] Abdulwadoud Alshoabi S, Noman Aljaber N, Omer Hussain A, Mohammed Aloufi K, Gafar Salih S (2020). Khat chewing effect on the international normalized ratio in patients with mechanical heart valves under warfarin therapy. Pakistan Journal of Biological Sciences : PJBS.

[45] Al-Motarreb AL, Broadley KJ (2003). Coronary and aortic vasoconstriction by cathinone, the active constituent of khat. Autonomic & Autacoid Pharmacology.

[46] Gonin P, Beysard N, Yersin B, Carron P-N (2018). Excited delirium: A systematic review. Academic Emergency Medicine : Official Journal of the Society for Academic Emergency Medicine.

[47] Zeppenfeld K, Tfelt-Hansen J, de Riva M, Winkel BG, Behr ER, Blom NA, Charron P, Corrado D, Dagres N, de Chillou C, Eckardt L, Friede T, Haugaa KH, Hocini M, Lambiase PD, Marijon E, Merino JL, Peichl P, Priori SG, Group, E. S. C. S. D (2022). 2022 ESC guidelines for the management of patients with ventricular arrhythmias and the prevention of sudden cardiac death: Developed by the task force for the management of patients with ventricular arrhythmias and the prevention of sudden cardiac death. European Heart Journal.

[48] Byrne RA, Rossello X, Coughlan JJ, Barbato E, Berry C, Chieffo A, Claeys MJ, Dan G-A, Dweck MR, Galbraith M, Gilard M, Hinterbuchner L, Jankowska EA, Jüni P, Kimura T, Kunadian V, Leosdottir M, Lorusso R, Pedretti RFE, Ibanez B (2023). 2023 ESC guidelines for the management of acute coronary syndromes: Developed by the task force on the management of acute coronary syndromes of the European Society of Cardiology (ESC). European Heart Journal.

[49] Beck O, Franzen L, Bäckberg M, Signell P, Helander A (2015). Intoxications involving MDPV in Sweden during 2010–2014: Results from the STRIDA project. Clinical Toxicology (Philadelphia, Pa.).

[50] Beck O, Franzén L, Bäckberg M, Signell P, Helander A (2016). Toxicity evaluation of α-pyrrolidinovalerophenone (α-PVP): results from intoxication cases within the STRIDA project. Clinical Toxicology (Philadelphia, Pa.).

[51] Carbone PN, Carbone DL, Carstairs SD, Luzi SA (2013). Sudden cardiac death associated with methylone use. The American Journal of Forensic Medicine and Pathology.

[52] Cawrse BM, Levine B, Jufer RA, Fowler DR, Vorce SP, Dickson AJ, Holler JM (2012). Distribution of methylone in four postmortem cases. Journal of Analytical Toxicology.

[53] Chou H-H, Hsieh C-H, Chaou C-H, Chen C-K, Yen T-H, Liao S-C, Seak C-J, Chen H-Y (2021). Synthetic cathinone poisoning from ingestion of drug-laced ‘instant coffee packets’ in Taiwan. Human & Experimental Toxicology.

[54] Desharnais B, Dazé Y, Huppertz LM, Mireault P, Skinner CD (2017). A case of fatal idiosyncratic reaction to the designer drug 3,4-methylenedioxypyrovalerone (MDPV) and review of the literature. Forensic Science, Medicine, and Pathology.

[55] Fujita Y, Koeda A, Fujino Y, Onodera M, Kikuchi S, Niitsu H, Iwasaki Y, Usui K, Inoue Y (2016). Clinical and toxicological findings of acute intoxication with synthetic cannabinoids and cathinones. Acute Medicine & Surgery.

[56] Hobbs JM, DeRienz RT, Baker DD, Shuttleworth MR, Pandey M (2022). Fatal Intoxication by the Novel Cathinone 4-Fluoro-3-methyl-α-PVP. Journal of Analytical Toxicology.

[57] Ikeji C, Sittambalam CD, Camire LM, Weisman DS (2018). Fatal intoxication with N-ethylpentylone: a case report. In Journal of Community Hospital Internal Medicine Perspectives.

[58] James D, Adams RD, Spears R, Cooper G, Lupton DJ, Thompson JP, Thomas SHL (2011). Clinical characteristics of mephedrone toxicity reported to the UK National Poisons Information Service. Emergency Medicine Journal: EMJ.

[59] Kesha K, Boggs CL, Ripple MG, Allan CH, Levine B, Jufer-Phipps R, Doyon S, Chi P, Fowler DR (2013). Methylenedioxypyrovalerone (‘bath salts’), related death: Case report and review of the literature. Journal of Forensic Sciences.

[60] Lee P-Y, Hsu C-C, Chan C-H (2022). Synthetic cathinone-induced myocarditis and psychosis: A case report. Journal of Addiction Medicine.

[61] Liveri K, Constantinou MA, Afxentiou M, Kanari P (2016). A fatal intoxication related to MDPV and pentedrone combined with antipsychotic and antidepressant substances in Cyprus. Forensic Science International.

[62] Murray BL, Murphy CM, Beuhler MC (2012). Death following recreational use of designer drug ‘bath salts’ containing 3,4-Methylenedioxypyrovalerone (MDPV). Journal of Medical Toxicology : Official Journal of the American College of Medical Toxicology.

[63] Nakamura M, Takaso M, Takeda A, Hitosugi M (2022). A fatal case of intoxication from a single use of eutylone: Clinical symptoms and quantitative analysis results. Legal Medicine.

[64] Nicholson PJ, Quinn MJ, Dodd JD (2010). Headshop heartache: Acute mephedrone ‘meow’ myocarditis. Heart (British Cardiac Society).

[65] Sellors K, Jones A, Chan B (2014). Death due to intravenous use of α-pyrrolidinopentiophenone. The Medical Journal of Australia.

[66] Sivagnanam K, Chaudari D, Lopez P, Sutherland ME, Ramu VK (2013). ‘Bath salts’ induced severe reversible cardiomyopathy. The American Journal of Case Reports.

[67] Weng T-I, Chen H-Y, Chin LW, Chou H-H, Wu M-H, Chen G-Y, Chen J-Y, Shih C-P, Lin C-C, Fang C-C (2022). Comparison of clinical characteristics between meth/amphetamine and synthetic cathinone users presented to the emergency department. Clinical Toxicology (Philadelphia, Pa.).

