# Supplementary material for: Cardiotoxicity After Synthetic Cathinone Use; Two Cases, A Case Series and Scoping Review
Source: Cardiovasc Toxicol. 2024 Feb 27;24(3):209–24. doi: 10.1007/s12012-024-09832-x (PMC10937789; doi:10.1007/s12012-024-09832-x)
Supplement: Supplementary file 1 — Supplementary file1 (DOCX 658 KB) [file 12012_2024_9832_MOESM1_ESM.docx]

**SUPLEMENTARY INFORMATION (SI)**

**SI 1** Pubmed search

**("1-phenyl-2-(1-pyrrolidinyl)-1-pentanone" [Supplementary Concept] OR flakka[tiab] OR α-PVP[tiab] OR alpha-pyrrolidinopentiophenon*[tiab] OR alpha-pyrrolidinovalerophenon*[tiab] OR "alpha-pyrrolidinovalerophenone" [Supplementary Concept] OR alpha-PVP[tiab] OR alphapyrrolidinopentiophenon*[tiab] OR alphapyrrolidinovalerophe-non*[tiab] OR zombie-drug*[tiab] OR gravel[tiab] OR "2-(4-bromo-2,5-dimethoxyphenyl)-N-((2-methoxyphenyl)methyl)ethanamine" [Supplementary Concept] OR NBOM*[tiab] OR bath-salt*[tiab] OR "mephedrone" [Supplementary Concept] OR mephedron*[tiab] OR 4-methylmethcathinon*[tiab] OR meow-meow[tiab] OR miaow[tiab] OR 4-MMC[tiab] OR MMCAT[tiab] OR "methylone" [Supplementary Concept] OR methylon*[tiab] OR 4-methycathinon*[tiab] OR n-ethylcathinon*[tiab] OR bk-MDMA[tiab] OR "3,4-methylenedioxypyrovalerone" [Supplementary Concept] OR MDPV[tiab] OR methylenedioxypy-rovaleron*[tiab] OR 3-methylmethcathinon*[tiab] OR 3-MMC[tiab] OR methaphedron*[tiab] OR 3-methylethcathinon*[tiab] OR 3-MEC[tiab] OR 4-methylethcathinon*[tiab] OR 4-MEC[tiab] OR ("4-methylethcathinone" [Supplementary Concept]) OR 3-chloromethcathinon*[tiab] OR 3-CMC[tiab] OR 4-chloromethcathinon*[tiab] OR 4-CMC[tiab] OR "clephedrone" [Supplementary Concept] OR 4-chlorodimethcathinon*[tiab] OR 4-CDC[tiab] OR 3-chloroethcathinon*[tiab] OR 3-CEC[tiab] OR 4-chloroethcathinon*[tiab] OR 4-CEC[tiab] OR mexedron*[tiab] OR "mexedrone" [Supplementary Concept] OR pentedron*[tiab] OR "pentedrone" [Supplementary Concept] OR N-ethylpentedron*[tiab] OR 4-FPD[tiab] OR hexedron*[tiab] OR N-ethylhexedron*[tiab] OR alpha-pyrrolidinoisohexaphenon*[tiab] OR α-PiHP[tiab] OR α-PHP[tiab] OR α-Pyrrolidinohexanophenon*[tiab] OR "alpha-pyrrolidinohexanophenone" [Supplementary Concept] OR α-PPP[tiab] OR alpha-pyrrolidinopropiophenon*[tiab] OR "alpha-pyrrolidinopropiophenone" [Supplementary Concept] OR cathinon*[tiab] OR "cathinone" [Supplementary Concept] OR New-psychoactive*[tiab] OR novel-psychoactive*[tiab]) AND ("Heart Diseases"[Mesh] OR cardiotox*[tiab] OR cardiac[tiab] OR cardial[tiab] OR heart[tiab] OR myocardi*[tiab] OR coronary[tiab] OR arrhythmi*[tiab] OR hypertensi*[tiab] OR tachycard*[tiab] OR STEMI[tiab] OR NSTEMI[tiab])**

**SI 2** Screening and selection tool for the inclusion and exclusion criteria and process

**1. Is the article about synthetic cathinones (including street names currently known)?**

No -> STOP. Excluded (Not relevant to topic)

Yes -> Proceed to 2.

**2. Is the article about a severe cardiac complication (including with supraventricular and ventricular arrhythmia’s, myocarditis, cardiomyopathy, acute coronary syndrome or cardiac arrest)?**

No -> STOP. Excluded (Not relevant to topic)

Yes -> Proceed to 3.

**3. Is the article of any following study designs or publication types?**

- Animal studies
- Lab studies
- Reviews
- Editorials / Letters to the editor
- Congress related

No -> Proceed to 4.

Yes-> STOP. Excluded (Excluded by study design or publication type)

**4. Is the article published in English or Dutch?**

No -> STOP. Excluded (Excluded study language)

Yes-> Proceed to 5.

**5. Does the title/abstract (stage 1) or study (stage 2) describe cardiac arrest or death due to other causes (including hyperthermia, seizures, renal failure, multi organ failure, serotonergic syndrome, agitation)?**

No -> STOP. Excluded (Excluded by no correlation)

Yes-> Proceed to 6.

**6. Does the title/abstract (stage 1) or study (stage 2) report coingestion of amphetamines, amphetamine-analogues, heroine, GHB, and/or cocaine?**

No -> STOP. Excluded (Excluded co-intoxication; no clear correlation)

Yes-> Proceed to 7.

**7. Does the title/abstract (stage 1) or study (stage 2) describes cases with self-reported and/or toxicologically confirmed synthetic cathinone?**

No -> Exclude

Yes -> Include!

**SI 3** Flowchart of study selection

PubMed database search

N = 363

Included based on title

N = 133

Excluded based on title

N = 230

Excluded based on abstract

N = 57

Included based on abstract

N = 76

Excluded based on full text

N = 46

Main reasons for exclusion:

N = 26 no cardiac event
N = 1 ECG abnormalities but normal lab

N = 5 review

N = 3 different subject

N = 2 different research question

N = 3 no specification between drugs

N = 1 language [Spanish]

N = 2 letter to the editor

N = 3 co-intoxication cocaine/heroine/MDMA

Included based on full text

N = 30

**SI 4** Etiquette of *Alegria Forest Fruit*


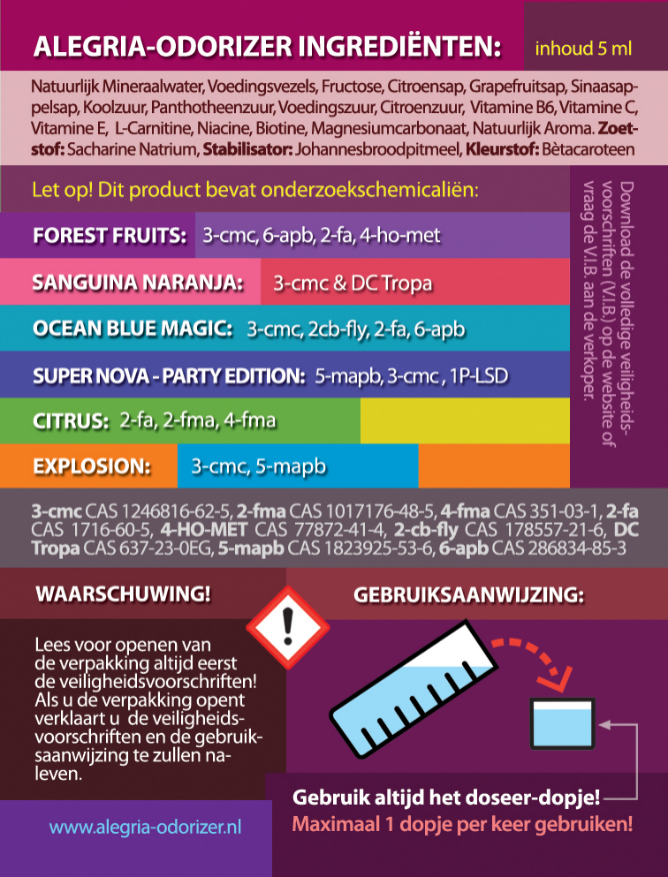


| **SI 5** High sensitive troponin-T time table case 1 | | | | | |
| --- | --- | --- | --- | --- | --- |
| **Time** | *referential* | 21:58 | 22:46 | 00:15 | 01:30 |
| **T (h)** |  | 0 | 1 | 2 | 3.5 |
| **Hs-troponin T** | *< 14 ng/l* | 16 | 21 | 21 | 20 |
| **CK-MB** | *< 7.6 µg/l* | 1.2 | 1.4 | 1.5 | 1.7 |
| *Abbreviations: h = hour, Hs = high sensitive, CK-MB = creatine kinase – myocardial band, ng/l = nanogram per liter, µg/l = microgram per liter.* | | | | | |

| **SI 6** Venous blood gas analysis case 1 | | |  |
| --- | --- | --- | --- |
|  | 21:58 | *Referential* | |
| Sodium | 138 | *135-147 mmol/L* | |
| Potassium | 3.7 | *3.5-5.0 mmol/l* | |
| Chloride | 109 | *96.0-109.0 mmol/l* | |
| Ca.ion.(pH 7,4) | 1.21 | *1.10-1.30 mmol/l* | |
| Glucose | 10.8 | *4.0-7.8 mmol/l* | |
| pH | 7.53 | *7.31-7.41* | |
| pCO2 | 25 | *41-51 mmHg* | |
| pO2 | 43 | *30-40 mmHg* | |
| Bicarb | 21.0 | *23.0-29.0 mmol/l* | |
| Base excess | 0.1 | *-3.0-3.0 mmol/l* | |
| O2 saturation | 87.0 | *>75.0 %* | |
| Lactate | 4.6 | *0.5-1.7 mmol/l* | |

| **SI 7** High sensitive troponin-T time table case 2 | | | | | |
| --- | --- | --- | --- | --- | --- |
| **Time** | *Referential* | 20:24 | 23:47 | 05:36 | 11:40 |
| **T (h)** |  | 0 | 3 | 9 | 3.5 |
| **Hs-troponin T** | *< 14 ng/l* | 48 | 837 | 475 | 285 |
| **CK-MB** | *< 7.6 µg/l* | 5.8 | 17 | 18 | 14 |
| *Abbreviations: h = hour, Hs = high sensitive, CK-MB = creatine kinase – myocardial band, ng/l = nanogram per liter, µg/l = microgram per liter.* | | | | | |

| **SI 8** Arterial blood gas analysis case 2 | | |
| --- | --- | --- |
|  | 20:26 | *Referentials* |
| Sodium | 141 | *135-147 mmol/L* |
| Potassium | 4.4 | *3.5-5.0 mmol/l* |
| Glucose | 8.5 | *4.0-7.8 mmol/l* |
| pH | 7.26 | *7.35-7.45* |
| pCO2 | 52 | *35-44 mmHg* |
| pO2 (mmHg) | 23 | *70-100 mmHg* |
| Bicarb | 23.1 | *22.0-29.0 mmol/l* |
| Base excess | -5.0 | *-3.0-3.0 mmol/l* |
| O2 saturation | 31.0 | *95.0-98.0 %* |

| Specific substances | Cardiotoxicity N = 288 |
| --- | --- |
| 1 | 3-MMC, 4-MMC |
| 2 | 3-MMC, 4-MMC |
| 3 | 3-MMC, 4-MMC |
| 4 | 3-MMC, 4-MMC |
| 5 | 3-MEC, 4-CMC |
| 6 | 3-MMC, 4-CMC (Alegria forest fruits) |
| 7 | 3-MMC, 4-CMC (Alegria forest fruits) |
| 8 | alpha-PiHP, 3-MMC or 4-MMC |
| 9 | alpha-PiHP, alpha-PCYP |
| 10 | 3-MMC, 4-CMC (Alegria forest fruits) |
| 11 | alpha-PiHP, 3-MMC |
| 12 | 3-MMC, 3-CMC or 4-CMC |
|  | |

**SI 9** Exposure to two synthetic cathinones

| SI 10 Case description of included cases presented in table 5 | | |
| --- | --- | --- |
| Author | Country | Case description |
| Beck et al.,  2015 [37] | Sweden | Not specified. |
| Beck et al.,  2016 [38] | Sweden | Unknown age and gender patient was in an agitated and delirious state and went into cardiac arrest. After 25 minutes of cardiopulmonary resuscitation ROSC was obtained, however the patients subsequently suffered from MOF and died after 4 days hospitalization. |
| Carbone et al.,  2013 [39] | Canada | 19-year old male collapsed while jogging without bracing himself. Witnesses reported him to be unresponsive, pulseless and apnoeic and started cardiopulmonary resuscitation. The paramedics arrived after five minutes, and the first rhythm on the cardiac monitor was PEA. Cardiopulmonary resuscitation and defibrillation was unsuccessful. He was pronounced dead after arriving at the ED. |
| Cawrse et al.,  2012 [40] | USA | 19-year old male collapsed while running during a physical fitness test. CPR was performed. After unknown time the patient CPR was stopped and the patients was pronounced dead. |
| Cherry and Rodriguez,  2017 [26] | USA | 41-year old female presented to the ED with an anterior STEMI after ingesting α-PVP. CAG revealed a proximal thrombus of the LAD and 80-90% stenosis of proximal ramus intermedius. Also there was a left ventricular apical thrombus. She was admitted to the ICU for respiratory failure due to pulmonary oedema. Thrombophilia workup was negative. She was discharged home after a lengthy hospital stay. |
| Chou et al.,  2021 [41] | Taiwan | 37-year old male ingested toxic coffee packets and unknown sedatives, after which he developed agitation, seizure and VT. Het was admitted to the ICU and died from the poisoning. |
| deRoux and Dunn,  2016 [27] | USA | 30 year old male who experienced a sudden collapse following physical altercation |
|  |  | 25 year old female with a history of hyperthyroidism on propranolol, was ‘oud partying’. She complained of a headache when returning home and was found unresponsive in bed. She was resuscitated and survived 10 hours in the hospital. Postmortem femoral blood ethylone concentration was 1.7mg/L and propranolol concentration was within therapeutic range. |
|  |  | 32 year old male was found dead at home with methylone heart blood concentration of 0.64mg/L |
|  |  | Two fatalities of a 37 and 42 year old male who were found dead with postmortem complicating atherosclerotic cardiovascular disease with coronary artery thrombosis (not specified how detected). |
| Desharnais et al.,  2017 [42] | Canada | 42-year old male presented with hysterical and disorganized behaviour in his prison cell, upon he was restrained. 27 minutes later he was in cardiorespiratory arrest and died after 1h40 minutes resuscitation with a body temperature of 39.3 degrees Celsius. Autopsy revealed left pleural adhesions and cardiomegaly with slight left ventricular hypertrophy. |
| Eiden et al.,  2013 [12] | France | 32-year old male expressed feeling ill after snorting PVP. He developed peripheral cyanosis, difficulty breathing and consequently lost consciousness. Cardiopulmonary resuscitation was performed on the scene by witnesses and later by emergency medical services, but was unsuccessful. |
| Froberg et al.,  2015 [23] | USA | SVT, not specified. |
| Fujita et al.,  2016 [43] | Japan | 23-year old male, ingested drugs in the restroom, after which he fell into bed and fell asleep. He was found with respiratory signs and was transferred to the hospital, where he experienced cardiopulmonary arrest on arrival. At autopsy no other causes of death were found except for heart failure. |
| Hobbs et al.,  2022 [44] | USA | 30-year old male was found unresponsive. Upon medical services arrival, first rhythm was VT upon which the man was defibrillated. He was then intubated and brought into the ED in cardiac arrest, after which he was declared death. |
| Ikeji et al.,  2018 [45] | USA | 21-year old male was combative, confused and sweating heavily. 5mg intramuscular haloperidol was administered, after which the patient went into cardiac arrest. ROSC was established after 3 minutes of cardiac massage, no rhythm check was performed. The first ECG showed a normal QTc. The patient was admitted to the ICU with a severe metabolic acidosis, and died after 72 hours due to multi organ failure. |
| James et al.,  2011 [46] | UK | 1. Chest pain and ECG-changes suggesting acute myocardial infarction. No further information. |
|  |  | 2. Generalised convulsions followed by apparent cardiac arrest. No further information. |
|  |  | 3. Cardiac arrest after mephedrone use, with unsuccessful resuscitation. No further information. |
| Janiszewski et al.,  2015 [28] | Poland | 26-year old male, with a history of hypertension and smoking, presented with retrosternal chest pain after ingestion of ethanol and insufflation of mephedrone. ECG showed ST-elevation in V2-V5. CAG revealed a total occlusion of the mid-LAD, for which he received percutaneous coronary intervention with a drug-eluting stent. The result was good. Echocardiography afterward revealed hypokinesis of the anterior wall with an LV EF of 55%. The patient was discharged in good condition. |
| Kesha et al.,  2013 [47] | USA | 37-year old male was brought in delusional and agitated after having used bath salts. Due to progressive agitation he was sedated and intubated at the ICU. He developed VT for which amiodorone and defibrillation was performed. Subsequently he became hypertherm (107 kelvin) and became bradycardic (36bpm). Despite atropine and external pacing he went into asystole with no effects of further cardiac resuscitation. |
| Kovács et al.,  2019 [29] | Hungary | 23-year old male felt sick after having used ‘crystal’. He was kneeling on the bed, then leaned forward and collapsed. Resuscitation by the ambulance was unsuccessful. History revealed a heart murmur with a sinus arrhythmia at the age of 10. Also he had seizures multiple times, with idiopathic generalized epilepsy-related signals on the EEG. Autopsy revealed hypertrophic and dilated cardiomyopathy, severe atherosclerosis of the valves, coronaries and arteries, and edema of the internal organs. |
| Lee et al.,  2022 [48] | Taiwan | 30-year old woman experienced chest pain, cold sweats and dyspnoea one morning after having used ‘instant coffee packets’ and inhalation of nitrous-oxide the night before. She was used to the coffee packets 2 to 3 times a week. At the ED her ultrasound showed global hypokinesis with an EF of 20% and a 1.5cm-sized suspicious vegetation in the left ventricle. He was admitted for treatment for myocarditis or suspected bacterial endocarditis, and suspected myocardial infarction. Follow-up ultrasound 3 days after starting treatment showed an EF of 33.7%, moderate mitral and tricuspid valve regurgitation, mild pulmonic regurgitation, and left ventricle diastolic dysfunction were also seen, all of which were indicative of dilated cardiomyopathy and myocarditis. Myocardial perfusion imaging with thallium-201 showed mild inducible ischemia at the apex, apical to mid-anterior and to mid-septal wall; mild ischemia or diaphragmatic attenuation at the basal inferolateral wall; a dilated left ventricle; right ventricle overload; poor left ventricle contractility; and stress-induced left ventricle dysfunction. |
| Lenz et al.,  2013 [24] | USA | 22-year old male was brought in the ED with syncope and confusion after having insufflated “cristalius”, which is (Mephedrone). He was observed at the CCU due to elevated cardiac enzymes (0.516ng/mL) and elevated creatinine kinase (668 U/L), with normal ECG and telemetry overnight. Troponins decreased to normal, CK elevated to 1544. With no physical complaints he was discharged. |
| Liveri et al.,  2016 [49] | Cyprus | 42-year old male, with known psychiatric illness, was found unconscious in his room. Cardiopulmonary resuscitation was unsuccessful. Autopsy revealed an hypertrophic enlarged heart, no significant atherosclerosis of coronary arteries. Ischemic areas in the anterior and posterior wall of the left ventricle and in the inter ventricular septum were observed. Cause of death was due to the concomitant intake of cathinones with CNS depressants that likely accounted for MI and fatal outcome. |
| Maskell et al.,  2011 [30] | UK | 49-year old female experienced chest pain, vomited and then collapsed after having insufflated mephedrone and having smoked cannabis. The EMS performed cardiopulmonary resuscitation, however unsuccessfully. Autopsy revealed an old atherosclerotic occlusion of the proximal anterior descending artery, with a 15mm diameter area of myocardial fibrosis within the anterior left ventricular wall near the apex. Histopathological examination disclosed additional diffuse fibrosis of the left ventricular myocardium, but no evidence of acute ischemia. |
|  |  | 19-year old male consumed mephedrone, alcohol and XTC after which he showed shaking and twitching, with abnormal behaviour and he was sweating. He was put into bed and shortly after, he was found unconscious with his eyes rolling and chocking. Cardiopulmonary resuscitation was unsuccessful. Autopsy disclosed no natural disease or trauma. |
|  |  | 55-year old female was found unresponsive in bed the morning after taking unknown drugs and alcohol. A witness described her as having slow and heavy breathing. Her medical history revealed psychiatric problems, drug abuse and drug overdoses. Resuscitation was unsuccessful. Autopsy revealed atherosclerosis. Autopsy revealed focal single artery coronary atherosclerosis with no other natural disease or trauma. |
| Murray et al.,  2012 [50] | USA | 40-year old male snorted and injected bath salts, after which he developed aggressive and delusional behaviour. He was shocked by a control device three times. At ED arrival the ECG showed ST. Subsequently he developed bradycardia and consequently cardiac arrest with PEA. ROSC was obtained after 30 minutes of cardiopulmonary resuscitation. The patient died eventually of multi organ failure and was declared braindead. |
| Nakamura et al.,  2022 [51] | Japan | 32-year old male was running around in his underwear, shouting, and rolling on the ground. After, he was sitting, not able to communicate, after which he lay down prone on the ground and was found unconscious 20 minutes after the first symptoms. He was in cardiac arrest, the first rhythm at the ambulance was PEA, later asystole at the ED. Resuscitation was unsuccessful. Autopsy revealed no cardiac thrombosis, and congested and edematous lungs. |
| Nicholson et al.,  2010 [52] | Ireland | 19-year old male presented with chest pain after eating plant food containing mephedrone. ECG showed ST-elevation and cardiac enzymes were elevated (1.19ug/L). The cardiac MRI showed anterolateral acute myocardial edema, consistent with acute myocardial inflammation. |
| Nugteren-van Lonkhuyzen et al.,  2022 [8] | The Netherlands | Retrospectively selected electronic Case Report Form. Unknown age and gender patient presented with agitation, hyperthermia (T 38.3 C/100.9 F), tachycardia (HR 120bpm), VF, cardiac arrest in the ambulance. Laboratory: increased CK (280 U/L). patient was presented to the ED, admitted to the intensive care unit, resuscitated, sedated with midazolam and intubated. No underlying heart conditions that might have caused VF were found. |
| Potocka-Banas et al.,  2016 [13] | Poland | 28-year old male presented with sudden cardiac arrest, after taking ‘crystal’. Resuscitation was successful, but accompanied by seizures and atrial fibrillation. He was admitted to the ICU where he was mechanically ventilated and received fluid therapy. Troponin T was 0.249ng/mL. The patient deteriorated with circulatory insufficiency, multiple episodes of cardiac arrhythmia requiring several defibrillation procedures. He went into a second cardiac arrest with no return of circulation after 5 hours of hospitalization. |
| Sellors et al.,  2014 [53] | Australia | 44-year old male injected himself with ‘smokin’ Slurry Scrubba’ after which he stripped of his cloths, jumped over a fence and smashed through a window. He was restrained to the floor, after which he suffered a cardiac arrest. CPR was performed by bystanders. On ambulance arrival the first rhythm was asystole. ROSC was obtained after 3 minutes and the total arrest time was 16 minutes. The patient was hypotensive (65/20) for which adrenalin perfusion was started. Eventually he developed DIC, renal failure and cerebral oedema with cerebral infarction. He was declared braindead 43 hours after the cardiac arrest. |
| Sivagnanam et al.,  2013 [54] | USA | 27-year old male was brought in with agitation, tachycardia (117bpm) and mild hypotension (90/60). He had no chest pain, dyspnoea or syncope. He had been injecting and inhaling a combination of MDPV/mephedrone a few hours before presentation. Because of persisting hypotension with suspicion of cardiogenic shock, a TTE was performed that revealed dilated cardiomyopathy with LVF 15-20% and global hypokinesia. CAG was negative for coronary artery disease. A few weeks later the patient was asymptomatic and a repeat echocardiogram showed a LVF of 52% with significant improvement in hypokinesia. |
| Sykutera et al., 2015 [14] | Poland | 28-year old male was brought to the ED in asystole. There was no return of circulation after 30 minutes cardiac resuscitation. Autopsy revealed pulmonary edema and moderately advanced atherosclerotic lesions of the arteries. Microscopic observation revealed chronic changes in the heart (uneven staining pattern of the myocardium, fragmentation and waviness of fibers, perivascular connective tissue growth, intramuscular fibrosis and scarring, disintegration of cardiomyocytes, nuclear disintegration, loss of cross-striations and thickening of blood vessel walls) and the presence of hemosiderin-laden pulmonary macrophages (heart failure cells). |
| Weng et al.,  2022 [55] | Taiwan | Out of 87 retrospectively selected patients presented at the ED in Taiwan after having used synthetic cathinone, 3 (3.4%) presented with OHCA of which all three had return of spontaneous circulation. It is unknown which cathinone and if the patients survived. |
| *Abbreviations: ARDS: Adult Respiratory Distress Syndrome, C: Celsius, CAG: coronary angiogram, CCU: cardiac care unit, CK: creatinine kinase, CPR: cardiopulmonary resuscitation, COPD: chronic pulmonary obstruction disorder, DIC: diffuse intravascular coagulation, ECG: electrocardiogram, ED: emergency department, EMS: event medical services, GSC: Glasgow coma Scale, F: Fahrenheit, HIV: Human Immunodeficiency Virus, ICU: intensive care unit, MOF: multi organ failure, OHCA: out of hospital cardiac arrest, LAD: left anterior descending artery, LVF: left ventricle function, MDPV: 3,4-methylenedioxypyrovalerone, MI: myocardial infarction, MRI: magnetic resonance imaging, NT-proBNP: pro-brain natriuretic peptide, OHCA: out of hospital cardiac arrest, PEA: pulseless electrical activity, α-PVP: α-pyrrolidinovalerophenone, ROSC: return of spontaneous circulation, TTE: transthoracic echo, ST: sinus tachycardia, STEMI: ST-elevation myocardial infarction, SVT: supraventricular tachycardia, T: temperature, VF: ventricle fibrillation, XTC:* methylenedioxymethamphetamine | | |
|  | | |
